# Improved survival of porcine acute liver failure by a bioartificial liver device implanted with induced human functional hepatocytes

**DOI:** 10.1038/cr.2016.6

**Published:** 2016-01-15

**Authors:** Xiao-Lei Shi, Yimeng Gao, Yupeng Yan, Hucheng Ma, Lulu Sun, Pengyu Huang, Xuan Ni, Ludi Zhang, Xin Zhao, Haozhen Ren, Dan Hu, Yan Zhou, Feng Tian, Yuan Ji, Xin Cheng, Guoyu Pan, Yi-Tao Ding, Lijian Hui

**Affiliations:** 1 Department of Hepatobiliary Surgery, the Affiliated Drum Tower Hospital, Medical School of Nanjing University, Nanjing, Jiangsu, China; 2 State Key Laboratory of Cell Biology, Shanghai Institute of Biochemistry and Cell Biology, Shanghai Institutes for Biological Sciences, Chinese Academic of Sciences, Shanghai 200031, China; 3 School of Life Science and Technology, ShanghaiTech University, Shanghai, China; 4 Center for Drug Safety Evaluation and Research, Shanghai Institute of Materia Medica, Chinese Academy of Sciences, Shanghai, China; 5 State Key Laboratory of Bioreactor Engineering, School of Bioengineering, East China University of Science and Technology, Shanghai, China; 6 Department of Pathology, Zhongshan Hospital, Fudan University, Shanghai, China

**Keywords:** acute liver failure, functional hepatocytes, bioartificial liver

## Abstract

Acute liver failure (ALF) is a life-threatening illness. The extracorporeal cell-based bioartificial liver (BAL) system could bridge liver transplantation and facilitate liver regeneration for ALF patients by providing metabolic detoxification and synthetic functions. Previous BAL systems, based on hepatoma cells and non-human hepatocytes, achieved limited clinical advances, largely due to poor hepatic functions, cumbersome preparation or safety concerns of these cells. We previously generated human functional hepatocytes by lineage conversion (hiHeps). Here, by improving functional maturity of hiHeps and producing hiHeps at clinical scales (3 billion cells), we developed a hiHep-based BAL system (hiHep-BAL). In a porcine ALF model, hiHep-BAL treatment restored liver functions, corrected blood levels of ammonia and bilirubin, and prolonged survival. Importantly, human albumin and α-1-antitrypsin were detectable in hiHep-BAL-treated ALF pigs. Moreover, hiHep-BAL treatment led to attenuated liver damage, resolved inflammation and enhanced liver regeneration. Our findings indicate a promising clinical application of the hiHep-BAL system.

## Introduction

Acute liver failure (ALF), a disease with rapid development of hepatocellular dysfunction, carries a high risk of short-term mortality^1^. Evidence-based supportive care is very limited for ALF, and emergency liver transplantation is the only proven cure^2^. Due to the limited availability of donor liver organs, it is urgent to evaluate alternative therapies. Bioartificial liver (BAL) support system has been thus far developed to bridge liver transplantation or to facilitate liver regeneration with the aim of preventing severe complications caused by liver failure and improving survival^2,3,4,5,6,7,8,9,10,11,12,13,14,15^. Human hepatocytes, immortalized human hepatoma cell lines, and porcine hepatocytes have been proposed in BAL devices^2^. Human hepatocytes are the preferred cells for BAL devices, but to obtain sufficient human hepatocytes faces the same difficulty of organ shortage. Human hepatoma cell line C3A derived from HepG2 has been used in the ELAD^®^ extracorporeal liver support system by Vital Therapies, Inc. Although C3A cells produce human proteins, these cells are incompetent in metabolism and ammonia clearance^16^. Our previous study also showed that HepG2 cells are incapable of curing Concanavalin A-induced ALF in mice upon *in vivo* transplantation^17^. Porcine hepatocytes are close to human hepatocytes in terms of metabolism and ammonia elimination. However, to prepare fresh and high-quality porcine hepatocytes by liver perfusion is cumbersome when a BAL treatment is urgently needed for patients in the ICU. Besides, porcine hepatocytes have ethical concerns in some countries and regions and the potential risk of xenozoonosis and immunological response. Ideal cell sources for BAL would be human-derived cells that possess mature hepatic functions and the capability to readily expand in large quantities *in vitro*.

We have previously converted mouse and human fibroblasts into functional hepatocytes^17,18^. Human functional hepatocytes (hiHeps) induced by *FOXA3*, *HNF1A*, and *HNF4A* are expandable cells displaying functions characteristic of mature hepatocytes^17^. Upon *in vivo* transplantation in mice with liver diseases, these cells saved lives in around 30% of mice^17^. However, transplantation of hiHeps to human patients needs additional measures to reduce risk of potential tumorigenesis. To avoid safety concerns of *in vivo* transplantation and to bring hiHeps closer to clinical application, we characterized in this study whether hiHeps could be used in a BAL support system (hiHep-BAL) and tested hiHep-BAL specifically in treating large animals with ALF.

## Results

### Optimization and large-scale expansion of hiHep cells

**Figure 1:**
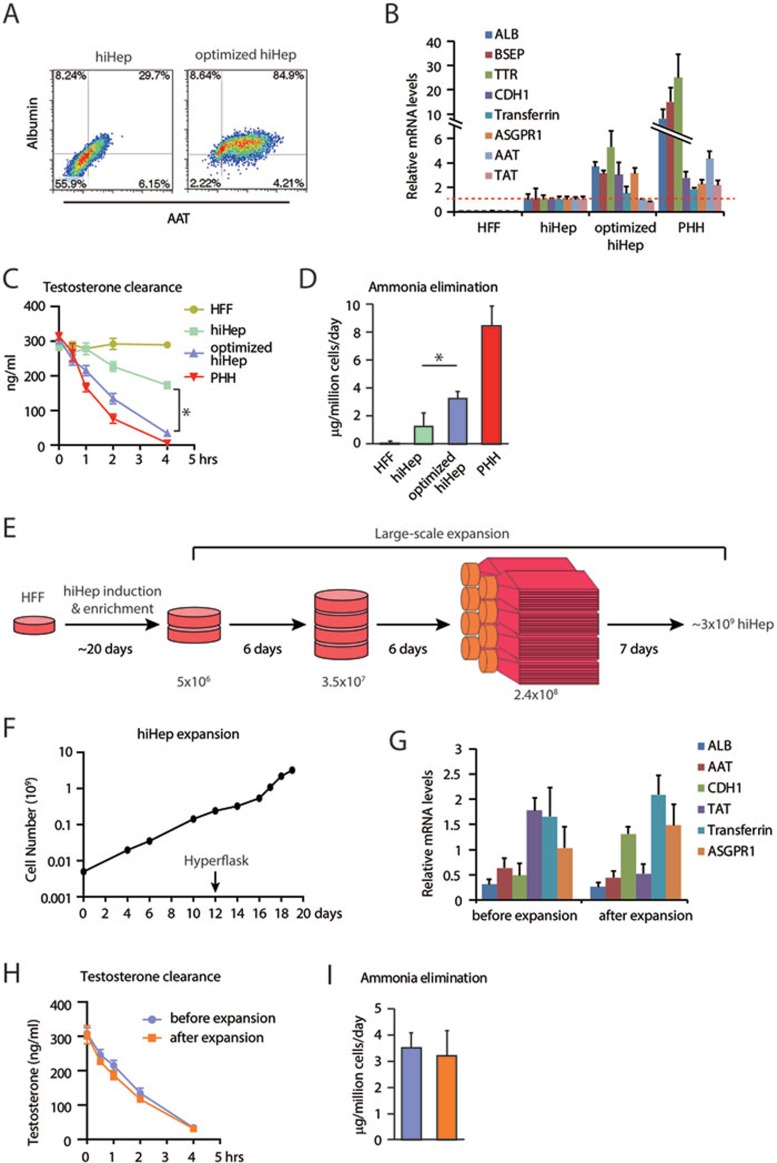
Characterization of optimized and large-scale expanded hiHeps. **(A)** Optimized hiHeps show high percentage of albumin and AAT double-positive cells as determined by flow cytometry. **(B)** q-PCR analyses of hepatic gene expression in optimized hiHeps, including albumin (ALB), ATP-binding cassette subfamily B member 11 (BSEP), transthyretin (TTR), cadherin 1 (CDH1), transferrin, asialoglycoprotein receptor 1 (ASGPR1), AAT, tyrosine aminotransferase (TAT). **(C)** Testosterone elimination of optimized hiHeps was determined by LC-MS/MS. **(D)** The ability of eliminating ammonia was measured in optimized hiHeps by enzymatic colorimetric assay. **(E)** Schematic outline of the large-scale expansion of hiHeps. hiHeps were generated and enriched in 6 cm petri dish and then expanded to 2.4 × 10^8^ in 10 cm dishes. hiHeps were finally expanded in Hyperflasks. **(F)** Large-scale expansion of hiHep cultures from 5 × 10^6^ to ∼3 × 10^9^ cells in 20 days. **(G**-**I)** Large-scale expanded hiHeps maintained hepatic gene expression levels **(G)**, efficient testosterone clearance **(H)** and ammonia elimination **(I)**. HFF, human fetal fibroblast. PHH, primary human hepatocyte. *P < 0.05, *t*-test.

First, we endeavored to further improve hepatic functions of hiHeps by optimizing the induction protocol, including fibroblast cultures, virus infection, medium components and passage procedures (Supplementary information, Figure S1A). Notably, a short treatment of collagenase during passage procedure enriched albumin and α-1-antitrypsin (AAT) double-positive hiHeps to over 80% in cultures (Figure 1A and Supplementary information, Figure S1B). Optimized hiHeps were validated in many functional aspects. First, they displayed homogeneous epithelial morphology and were positively stained for cadherin 1 (CDH1) and tight junction protein ZO-1 (Supplementary information, Figure S1B and S1C). Second, increased expression levels of hepatic genes were found in optimized hiHeps, while marker genes for immature hepatocytes were undetectable (Figure 1B and Supplementary information, Figure S2A). Moreover, optimized hiHeps acquired remarkable mature functions of albumin and AAT secretion, glycogen storage and ac-LDL intake compared with cultured primary human hepatocytes (PHHs; Supplementary information, Figure S2B–S2D). Optimized hiHeps also improved metabolic detoxification in several aspects, including increased testosterone elimination as indication of CYP3A function, enhanced bioactivation process of Troglitazone as formation of reactive metabolites and elevated biliary excretion as indices of xenobiotic elimination (Figure 1C, Supplementary information, Figure S2E and S2F). Importantly, optimized hiHeps possessed increased capability to eliminate ammonia (Figure 1D).

Another technical challenge for BAL application is to produce hiHeps at a large scale of 10^9^–10^10^ cells. We developed a strategy to scale up the size of the hiHep culture. Briefly, after propagating hiHeps to 2.4 × 10^8^ cells in 10 cm dishes, we expanded the culture in eight 1 720 cm^2^ Hyperflasks to obtain about 3 billion cells within 7 days (Figure 1E and 1F). During expansion, hiHep cultures were carefully monitored by measuring the pH values and nutrition component concentrations (Supplementary information, Figure S3A and data not shown). Expanded hiHep cells showed typical epithelial morphology without detectable aspartate aminotransferase (AST) leakage, indicating that the integrity of hiHeps is not impinged by large-scale and fast expansion (Supplementary information, Figure S3B and S3C). The percentage of the albumin and AAT double-positive cells were maintained during expansion (Supplementary information, Figure S3D). Also, the expanded hiHeps maintained high-level hepatic gene expression as well as hepatic functions in albumin secretion, testosterone elimination, and ammonia clearance (Figure 1G–1I, Supplementary information, Figure S3E). Among different batches of hiHep preparation, hepatic genes were expressed at comparable levels and consistent numbers of cells were harvested, indicating that the expansion procedure was robust and reproducible (Supplementary information, Figure S4A and S4B). hiHeps thus produced were applied in a homemade BAL support system^19,20,21,22,23^.

**Figure 2:**
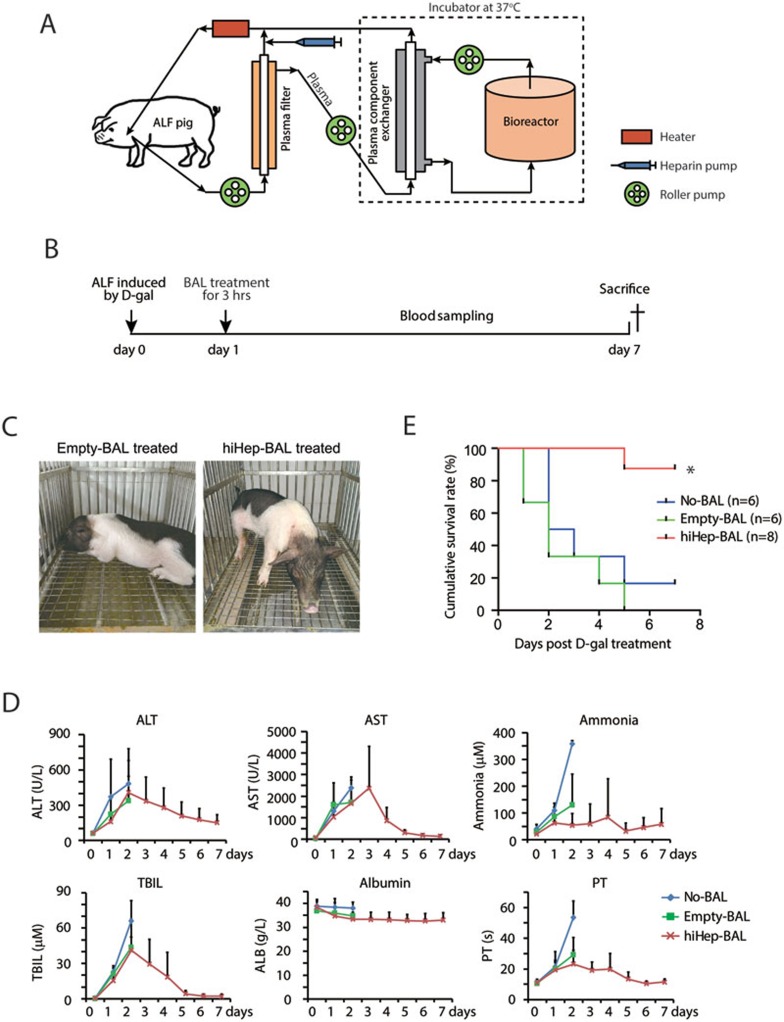
hiHep-BAL rescues ALF pigs. **(A)** Schematic diagram depicts the configuration of the hiHep-BAL support system. Approximately 3 × 10^9^ hiHeps were implanted into the bioreactor. **(B)** The outline of the hiHep-BAL treatment of ALF Bama miniature pigs. **(C)** Bama miniature pigs treated by Empty-BAL and hiHep-BAL. Note that ALF pigs after hiHep-BAL treatment were apparently active on day 5. **(D)** Serum biochemical parameters of ALF pigs in No-BAL, Empty-BAL and hiHep-BAL groups. Serum levels of ALT, AST, ammonia, TBIL, albumin and prothrombin time (PT) were measured. Because most animals of No-BAL and Empty-BAL groups were dead or extremely sick from day 3, blood samples were not collected in these two groups after day 3. **(E)** Kaplan-Meier survival curve of No-BAL, Empty-BAL- and hiHep-BAL-treated ALF pigs (*n* = 6 for No-BAL and Empty-BAL, *n* = 8 for hiHep-BAL group). *P < 0.01, log-rank test.

The homemade BAL support system consisted of a multi-layer radial-flow bioreactor, a plasma filter separating blood cells and blood plasma, a plasma component exchanger serving as immunoprotective barrier, and other accessories^19,20,21,22,23^ (Figure 2A). The fully assembled bioreactor contained a stack of 65-layer round flat plates, which were made of polycarbonate (Supplementary information, Figure S5A). Functional cells were perfused into the bioreactor and incubated until all cells adhered onto the polycarbonate plates^19,20,21,22,23^. We have previously demonstrated that this BAL support system implanted with primary porcine hepatocytes is effective and safe in treatment of ALF canines without leakage of cells or porcine endogenous retroviruses from the bioreactor^19,20,21^. We asked whether the hiHep-based BAL support system would show therapeutic effect on ALF pigs (Supplementary information, Figure S5B).

We first characterized whether hiHeps would adapt to conditions in the bioreactor by seeding them on the polycarbonate plates for 24 h. We found that hiHep cells attached to the polycarbonate surface efficiently (Supplementary information, Figure S6A) and maintained their integrity and maturation as shown by minimal AST leakage and proper cell membrane location of ASGPR1^24^, respectively (Supplementary information, Figure S6B and S6C). Compared with hiHeps cultured on collagen-coated petri dishes, hiHeps on the polycarbonate surface displayed well-maintained hepatic gene expression and albumin secretion within 24 h (Supplementary information, Figure S6D and S6E). Next, we confirmed whether hiHeps are resistant to toxic substances generated during the BAL treatment by culturing them with medium collected from bioreactor after BAL treatment of ALF pigs. The viability of hiHeps was not affected after exposure to the bioreactor medium for 24 h (Supplementary information, Figure S6F). Also, hiHeps maintained epithelial morphology and hepatic gene expression after bioreactor medium treatment (Supplementary information, Figure S6G and S6H). These data collectively indicate that hiHeps are compatible with polycarbonate surface and are tolerant to toxic substances during BAL treatment.

### Establishment of a ALF model in pigs

We induced ALF in adult Bama miniature pigs^25^ by intravenous injection of D-galactosamine (D-gal), a model recapitulating ALF features in humans (Figure 2B). To establish a proper ALF model, adult Bama miniature pigs were treated with D-gal at 0.35, 0.4, 0.45, and 0.5 g/kg body weight (Supplementary information, Figure S7A). D-gal doses at the comparable scale were used to induce ALF in other studies^26,27,28^. D-gal at 0.4 g/kg was chosen, because 0.35 g/kg D-gal-treated pigs survived 7 days, whereas 0.45 and 0.5 g/kg D-gal killed pigs within 2 days, which may cause a narrow treatment window (Supplementary information, Figure S7A). Importantly, on day 1 after 0.4 g/kg D-gal injection, Bama pigs developed severe ALF as manifested by apparent sickness and fatigue and vastly increased blood levels of alanine aminotransferase (ALT), AST, ammonia and total bilirubin (TBIL)^29^ (Supplementary information, Figure S7B). Because D-gal-induced ALF was severe and killed pigs quickly, we chose to perform BAL treatment 24 h after D-gal injection in order to provide sufficient treatment window. Notably, D-gal was efficiently eliminated from blood at this time point, excluding the possibility of direct D-gal detoxification by hiHep cells (Supplementary information, Figure S7C).

To choose cell numbers, we implanted 1, 1.5 and around 3 billion hiHeps into the bioreactor, respectively (Supplementary information, Figure S8A and S8B). Despite the small size of the experimental cohort, a strong correlation between survival time and implanted hiHep numbers was observed (Supplementary information, Figure S8A, Pearson correlation coefficient R^2^ = 0.98). Interestingly, we found that the weight of 3 billion hiHeps was about 30.9 g. Given that the liver/body weight ratio is 1.9% in adult Bama pigs^30^, 30.9 g of hiHep cells was roughly equivalent to 10% of total liver mass of a 15 kg pig. Taken together, we decided to induce ALF using 0.4 g/kg D-gal and to treat pigs with BAL implanted with 3 billion hiHeps on day 1 post D-gal administration (Figure 2B).

### BAL treatment of ALF pigs

Twenty adult Bama miniature pigs were divided into three groups and were induced to develop ALF by D-gal (Table 1). Pigs in the first group did not receive any treatment during the experiment (No-BAL group, *n* = 6). The second group of pigs was treated with the BAL support system containing no functional cells (Empty-BAL group, *n* = 6). The last group of pigs was hiHep-BAL-treated (hiHep-BAL group, *n* = 8). Pigs in all groups showed symptoms of hepatic encephalopathy after D-gal treatment, including fatigue, unresponsiveness and obvious drowsiness^29^. However, hiHep-BAL-treated pigs were apparently active on day 5, indicating improved recovery from D-gal-induced ALF (Figure 2C). Blood biochemical measurements showed that hiHep-BAL treatment already caused a decrease in ammonia levels and prothrombin time on day 2 after D-gal administration compared with No-BAL and Empty-BAL groups (Figure 2D). The decline tendency of ALT, AST, ammonia, TBIL levels and prothrombin time was already evident on day 3 in hiHep-BAL group and a prolonged benefit was observed in the hiHep-BAL group as demonstrated by correction of these blood biochemical parameters within 7 days (Figure 2D). Importantly, 7 out of 8 pigs survived in the hiHep-BAL group (Figure 2E, *P* < 0.01, log-rank test), which is comparable to porcine primary hepatocyte-based BAL support system^19,20,21,28^. By contrast, only 1 pig in the No-BAL group survived the D-gal-induced ALF, and all pigs died in the empty-BAL group (Figure 2E and Table 1). These data together indicate a remarkable therapeutic effect of the hiHep-BAL.

**Figure 3:**
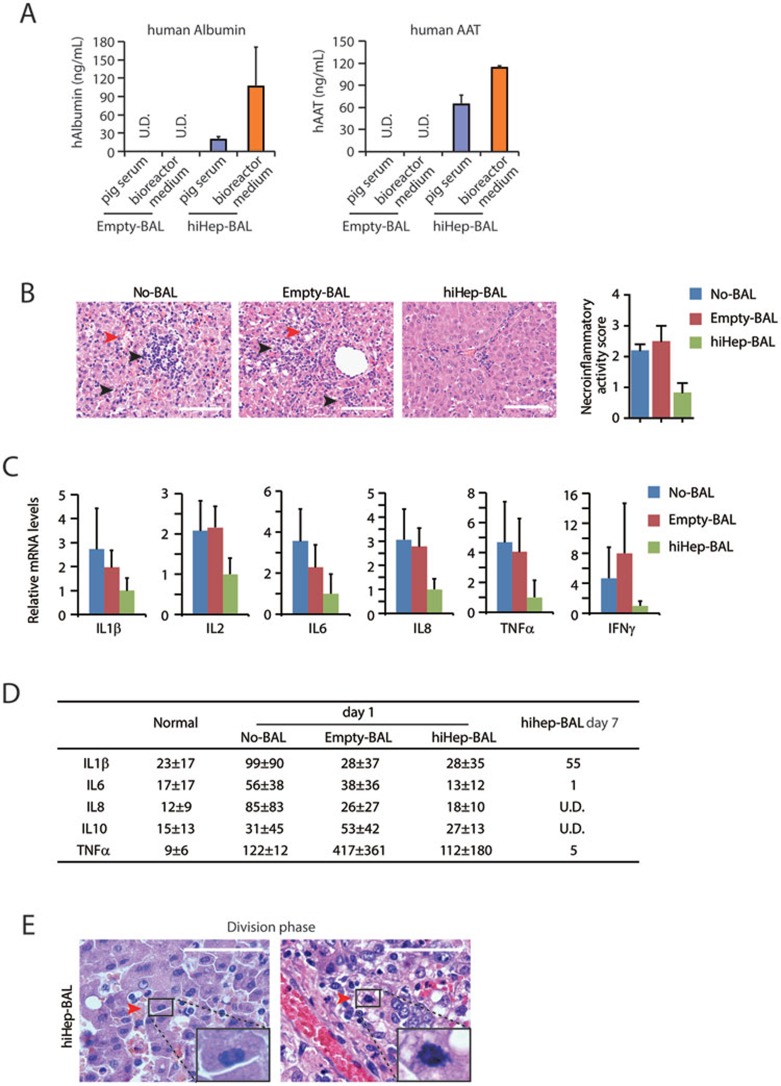
Therapeutic effects of hiHep-BAL on ALF pigs. **(A)** Human albumin and ATT were measured in the pig serum after Empty-BAL and hiHep-BAL treatment. Human protein-specific ELISA kit was used in the assay. **(B)** Hematoxylin and Eosin (HE) staining of pig livers of No-BAL, Empty-BAL and hiHep-BAL groups. Livers of No-BAL and Empty-BAL groups were collected on day 2 or 3 after D-gal induction. Livers of hiHep-BAL group were collected on day 7. Red arrowheads indicate liver damage, including karyorrhexis, karyolysis and hemorrhage. Black arrowheads indicate local infiltration of inflammatory cells. Necro-inflammation was scored according to the Scheuer system. Scale bar, 100 μm. **(C)** mRNA expression of inflammatory cytokine genes was determined by q-PCR in pig livers of No-BAL, Empty-BAL and hiHep-BAL groups, including IL1β, IL2, IL6, IL8, TNFα and IFNγ. **(D)** Serum levels of the indicated cytokines were determined by ELISA with antibodies specific for pig proteins. **(E)** Histological analyses of proliferating hepatocytes in hiHep-BAL-treated pigs on day 7. Red arrowheads indicate hepatocytes at division phase. High magnification images of division phase are inserted.

Next, we analyzed the therapeutic mechanism of hiHep-BAL on ALF. Markedly, human albumin and AAT were detectable in pig sera using ELISA assays with human-specific antibodies (Figure 3A). The measured human albumin and AAT levels (at the level of ng/mL) were at the same order of calculated values, if the amount of cells, treatment duration and total pig blood volume were taken into account. These data suggest that proteins and small-molecule substances synthesized and secreted by hiHeps could be infused into ALF pigs, providing an indication of the substance exchange in BAL therapeutic effect. D-gal triggered extensive cell death, and hemorrhage accompanied with massive inflammation in liver tissues of No-BAL and Empty-BAL groups on day 2 (Figure 3B and Supplementary information, Figure S9A). In the hiHep-BAL group, livers from pigs sacrificed on day 7 displayed markedly reduced tissue damages and cell death as shown by histological inspection, TUNEL staining and gene expression analyses (Figure 3B, Supplementary information, Figure S9A–S9C). The reduced tissue damages were associated with decreased infiltration of inflammatory cells in liver tissues of hiHep-BAL-treated pigs (Figure 3B and Supplementary information, Figure S9A). To confirm the attenuated inflammation, we measured levels of inflammation cytokines. ALF induced substantially increased expression of inflammation cytokines, such as TNFα and IFNγ, in No-BAL and Empty-BAL liver tissues 2 days after D-gal treatment (Figure 3C). mRNA levels of these cytokines were markedly downregulated in hiHep-BAL liver tissues on day 7 (Figure 3C). Accordingly, ELISA assays demonstrated that serum levels of inflammation cytokines showed a decrease tendency after BAL treatment and returned to normal levels on day 7 in hiHep-BAL group (Figure 3D). These data indicated the resolution of inflammatory response induced by ALF (Supplementary information, Figure S9D). Markedly, hiHep-BAL showed additional effects besides cytokine modulation. Along with reduced cell death and inflammation, liver tissues of hiHep-BAL group showed restored hepatic architectures (Figure 3B and Supplementary information, Figure S9A). Moreover, proliferating cells and genes involved in proliferation were still detectable in the hiHep-BAL group 7 days after injury (Figure 3E and Supplementary information, Figure S9D–S9F), suggesting improved liver regeneration. Collectively, these results show reduced liver cell death, resolved local inflammation and prominent tissue regeneration in hiHep-BAL-treated livers.

## Discussion

In this study, we developed a hiHep-based BAL support system. Although hiHeps were remarkably optimized in this study, it is worth mentioning that hiHeps are not the same as PHHs in all functional aspects. Nevertheless, hiHeps demonstrated therapeutic effect on improving survival of ALF pigs. Our data suggest that hiHep-BAL possesses notable potentials for metabolic detoxification by reducing serum levels of ammonia and TBIL and for synthetic activity by secreting human albumin and AAT. hiHep-BAL represents an advance for both hiHeps and the BAL support system in treating ALF^2,3^. Human hepatoma line C3A and porcine primary hepatocytes are the main functional cells currently proposed for the BAL system^2,3^. Compared to C3A cells, hiHep cells are more close to mature hepatocytes in metabolic detoxification and ammonia elimination. It is notable that ELAD, a HepG2 C3A-based BAL system, has recently failed phase III trial, further discouraging the use of HepG2 C3A in the BAL system. Compared to porcine primary hepatocytes that are cumbersome to prepare and need further test to minimize risks of xenozoonosis and immunological responses to porcine proteins^2,3,17,31^, hiHep cells are easily expanded to a large scale in a controlled manner and are human-derived cells. Because of these reasons and that the aim of this study was to validate the potential of hiHep cells for BAL, we did not include HepG2 C3A and primary porcine hepatocytes in our experiments. Moreover, an advantage of the BAL system is that it separates hiHeps from human tissues, therefore avoiding safety concerns regarding direct *in vivo* transplantation of hiHep cells^2,32^. hiHep cells thus represent a new source of human-derived functional cells, which largely solve the intrinsic limitations of hepatoma cells and porcine hepatocytes.

We have previously demonstrated the *in vivo* therapeutic effects of hiHep cells by transplanting them into mice with liver diseases^17^. Here we wished to show that hiHep cells could be used *in vitro* in BAL devices to treat ALF in large animals. The *in vivo* and *in vitro* microenvironments are very different for hiHep cells. Also, to scale up the culture brings additional uncertainty to the whole system. It is therefore not predictable prior to the experiment whether hiHep would show any benefit to ALF pigs. Interestingly, hiHep-BAL treatment showed a higher survival rate on ALF pigs (survival rate: 87.5%) compared to *in vivo* transplantation of hiHeps to mice with chronic liver metabolism diseases (survival rate: ∼30%). A likely explanation is that repopulation of transplanted cells is a low-efficiency process. Also in the ALF animals or patients, the remnant liver functions remained and the liver could regenerate after passing the critical point. To that end, we kept ALF pigs up to 7 days post ALF induction, which was longer than or as long as previous published studies on BAL-treated D-gal-induced large animals^9,19,28^. Extending survival time to 7 days provides ample opportunity for organ transplantation and liver regeneration from ALF. Our previous studies on dogs^19^ and current study on pigs further confirmed this.

Our findings suggest that hiHep-BAL could potentially save the lives of ALF patients. Several criteria need to be met before the clinical application of hiHep-BAL. For human treatment, over 10^10^ cells are required. By using an even larger cell culture system, such as HyperStack, it is feasible to expand hiHep cells to the number of > 10^10^. In addition, because hiHeps could be cryopreserved and resuscitated (data not shown), it is possible to pool different batches to achieve > 10^10^ cells. Also, it is important to further define the suitable population of patients and to develop prognostic measures post treatment^3^. It would be highly desirable to test whether hiHep-BAL could be applied to acute-on-chronic liver failure, the most frequent liver failure in clinics^33^. Apparently, ALF or acute-on-chronic liver failure in patients are more complex than D-gal-induced ALF in pigs, thus it remains uncertain whether hiHep-BAL would show therapeutic effect in patients. Finally, hiHep-BAL should be further improved for its clinical application, such as enhancing hiHep functions by co-culture with mesenchymal stem cells or endothelial cells^34,35,36^, and developing next generation of BAL support system by applying the spheroid reservoir BAL^26,28^. Nevertheless, given the remarkable effects of hiHep cells in treating ALF pigs and their readily and controllably expanding capability, it is worth testing hiHep-BAL in human ALF patients.

## Materials and Methods

### Cell culture and hiHep induction

Human fetal fibroblasts (HFFs) were cultured in human fibroblast medium (HFM). hiHep cells were cultured in collagen-coated dishes with hepatocyte maintenance medium (HMM). PHHs from 3 individuals were purchased from Celsis In Vitro Technologies (Lot#: TLQ, FLO, YJM). Detailed information about PHHs was provided in the product instructions. Institutional ethical committees approved collection and use of human samples.

### Large-scale expansion

After expansion in 100 mm dishes, final expansion of hiHep cells was performed in Hyperflasks (Corning). Finally, about 3 × 10^9^ hiHep cells were harvested from the Hyperflasks and used for the hiHep-BAL treatment.

### BAL system

The BAL system consists of a cell circuit and a blood circuit. The components of BAL system include three roller pumps, a heparin pump, a plasma filter (Sorin Group Italia, Mirandola, Italy), a plasma component separator (Kawasumi Laboratories Inc, Tokyo, Japan), and a multi-layer flat-plate bioreactor with polycarbonate scaffolds. The multi-layer bioreactor consists of a hollow column stent, and a stack of 65-layer round flat plates, all of which are made of polycarbonate.

### Miniature pig surgery

Chinese Bama miniature pigs (15-25 kg, 4-5 months of either sex)^25^ were purchased from the Laboratory Animal Center of the Affiliated Drum Tower Hospital of Nanjing University Medical School. All animal procedures were performed according to institutional and national guidelines and approved by the Animal Care Ethics Committee of Nanjing University and Nanjing Drum Tower Hospital.

Pigs in all groups were anesthetized by propofol (0.1-0.2 mg/kg/min) and intravenously injected with D-gal, and baseline blood sample was collected. Blood samples were collected before and after BAL treatment. And every day afterwards, pig was clinically observed and blood sampled till the animals were sacrificed on day 7 post D-gal injection which is the endpoint of the study. All animal livers were collected for Hematoxylin and Eosin (H&E), Ki67 staining and q-PCR at the end.

### Pharmacokinetic analysis

Three minipigs were anesthetized by propofol (0.1-0.2 mg/kg/min) and intravenously injected with D-gal at the dosage of 0.4 g/kg. 5 ml blood samples for determination of the D-gal concentration were taken before drug administration, and 3 ml at 0.25, 0.5, 1, 1.5, 2, 4, 6, 8, 10, 12, 24, 36, 48 h after drug administration. D-gal in the supernatant was injected for ultra-performance liquid chromatography coupled with tandem mass spectrometry (LC-MS/MS) analysis.

### RNA extraction and quantitative PCR

For most experiments, total RNA was isolated from cells by Trizol (Invitrogen). For RNA extraction from formalin-fixed paraffin-embedded (FFPE) tissues, RNA was extracted using RNeasy FFPE Kit (Qiagen). Quantitative PCR (qPCR) was performed with SYBR Premix Ex Taq (TaKaRa) on an ABI StepOne Plus real-time PCR system (Applied Biosystems).

### ELISA, assays for glycogen storage and intake of ac-LDL

To determine the secretion of human albumin and AAT, supernatants of cell culture were measured using human-specific Albumin ELISA Quantitation Set (Bethyl Laboratory) and human α-1-Antitrypsin ELISA kit (Bethyl Laboratory). The amounts of porcine IL1β, IL6, IL8, IL10 and TNFα in pig serum were measured using porcine-specific ELISA Quantitation Set (R&D Systems). Glycogen storage of hiHep, optimized hiHep, HFF and PHH was determined by Glycogen Assay Kit (Abnova). Cells were incubated with DiI-ac-LDL (Invitrogen) for 6 h, and then the intake of ac-LDL was measured quantitatively by Operetta (PerkinElmer).

### CYP metabolism and biliary excretion assay

For measurement of CYP metabolic activities, the supernatants were collected for measurement of the indicated productions by LC-MS/MS (LCMS-8030; Shimadzu, Kyoto, Japan). D8-TCA and Rosuvastatin were analyzed by LC/MS/MS. The amount of CLF was quantified by measuring fluorescence at 492 nm and 536 nm with a Synergy 4 microplate reader (Biotek, Winooski, USA). Biliary Excretion Index (BEI) was calculated as: BEI = (AHBSS – AHBSS(Ca^2+^ free))/AHBSS × 100%.

### Histology and immunohistochemistry

Liver tissues were collected immediately at the time when pigs died or were sacrificed, and fixed overnight with 4% neutral formalin. Tissue sections were stained with haematoxylin (Sigma-Aldrich) and eosin (Sigma-Aldrich) for pathological evaluation. Ki67 staining was performed according to the protocols described previously^17^. TUNEL staining was performed using the ApoAlertTM DNA fragmentation Assay Kit (BD Sciences).

### Statistics

For most statistic evaluation, an unpaired Student's *t*-test was applied to calculate statistical probability in this study. *P* values were calculated by two-tailed test. For survival analysis, the Mantel-Cox log-rank test was applied. Statistic calculation was performed using Statistical Program for Social Sciences software (SPSS, IBM). For all statistics, data from at least five biological replicates were used.

Detailed Materials and Methods are provided in Supplementary information, Data S1.

## Author Contributions

L Hui, Y-T Ding, G Pan and X-L Shi conceived and supervised the study. Y Gao, Y Yan, L Sun, P Huang and L Zhang optimized the hiHep culture and performed most of the experiments. X-L Shi, H Ma, X Zhao, H Ren and Y-T Ding performed BAL experiments. X Ni and G Pan characterized and analyzed the CYP metabolism, drug toxicity and pharmacokinetics experiments. D Hu and Y Zhou are involved in large-scale expansion of hiHep cells. F Tian and Y Ji performed histological analyses. Y Gao, Y Yan, L Sun and L Hui analyzed the data and wrote the paper with suggestions from other authors.

## Competing Financial Interests

The authors declare no competing financial interests.

## Figures and Tables

**Table 1:** BAL treatment of ALF pigs

Group	Pig weight (kg)	hiHep number	Survival time*
No-BAL	18	0	2 days
No-BAL	15	0	Survived
No-BAL	16	0	2 days
No-BAL	22	0	2 days
No-BAL	16	0	3 days
No-BAL	17	0	5 days
Empty-BAL	12	0	1 days
Empty-BAL	18	0	2 days
Empty-BAL	16	0	1 days
Empty-BAL	16	0	2 days
Empty-BAL	23	0	4 days
Empty-BAL	15	0	5 days
hiHep-BAL	17	2.8 × 10^9^	Survived
hiHep-BAL	18	3.5 × 10^9^	Survived
hiHep-BAL	17	4.0 × 10^9^	Survived
hiHep-BAL	15	3.1 × 10^9^	Survived
hiHep-BAL	15	3.0 × 10^9^	5 days
hiHep-BAL	15	3.4 × 10^9^	Survived
hiHep-BAL	15	2.6 × 10^9^	Survived
hiHep-BAL	15	3.5 × 10^9^	Survived

^*^All survived animals were sacrificed on day 7.

## Supplementary Information

Supplementary information, Figure S1 Optimization of hiHeps

Supplementary information, Figure S2 Optimized hiHeps show increased hepatic gene expression and functions

Supplementary information, Figure S3 Stability of hiHep cells during the large-scale expansion

Supplementary information, Figure S4 Consistency of large-scale-expanded hiHeps between different batches

Supplementary information, Figure S5 The BAL support system

Supplementary information, Figure S6 Stability of hiHeps in the bioreactor

Supplementary information, Figure S7 Establish the D-gal-induced ALF model in Bama miniature pigs

Supplementary information, Figure S8 Determine the cell number of hiHeps for hiHep-BAL

Supplementary information, Figure S9 Characterization of hiHep-BAL-treated ALF pigs

Supplementary information, Data S1 Supplemental Experimental Procedures
