# Supplementary material for: Improved survival of porcine acute liver failure by a bioartificial liver device implanted with induced human functional hepatocytes
Source: Cell Res. 2016 Jan 15;26(2):206–16. doi: 10.1038/cr.2016.6 (PMC4746613; doi:10.1038/cr.2016.6)
Supplement: Supplementary information, Figure S1 — Optimization of hiHeps [file cr20166x1.pdf]

A

	Previous protocol	Optimized protocol
Initiating fibroblasts	Immortalized fibroblasts at any passages	Immortalized fibroblasts at defined passages (P8-15)
Seeding density	1.75-2.0x10^5/6cm dish	1.5-2.0x10^5/6cm dish
Infection time point	24 hrs after seeding	Infection during seeding
MOI	1	1.25
Medium supplement	0.03g/L proline	0.3g/L proline
Passage interval	4 days	6 days
Replating density	1:3 - 1:5	3-5x10^5/6cm dish
Digestion enzyme	Trypsin	Trypsin and collagenase

B

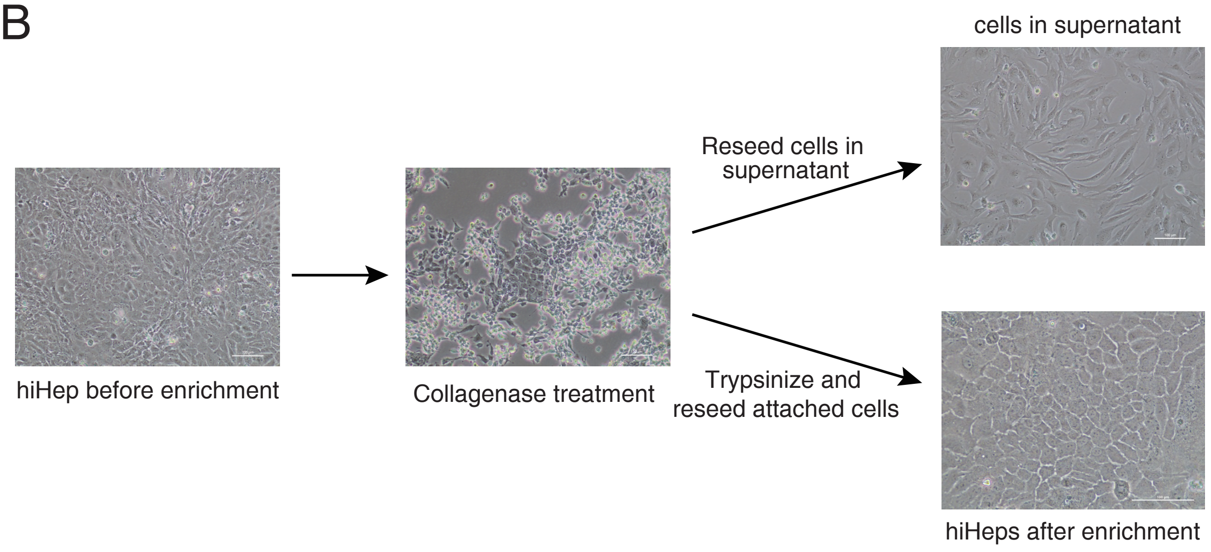

C

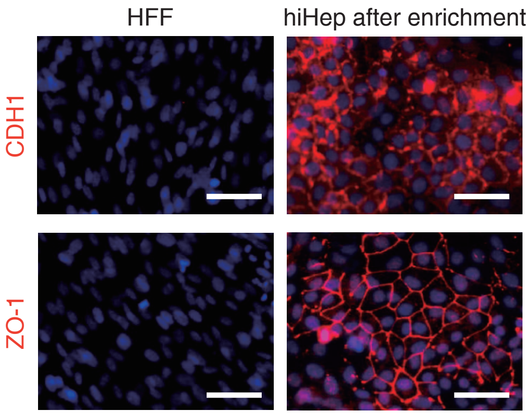

Supplemental Figure 1

Figure S1. Optimization of hiHeps

A, Summary of the improvements in the optimized protocol for hiHep induction and culture compared with the previous protocol. The optimized protocol has improvements in fibroblast culture, infection conditions, medium supplement and passage procedures. MOI, Multiplicity of infection. **B**, Strategy of hiHep enrichment by collagenase digestion. Enriched hiHep cells showed homogeneous epithelial morphology. Scale bar, 100 μm. **C**, Tight junction protein of hiHep cells measured by immunofluorescent staining of tight junction protein (ZO-1) and cadherin 1 (CDH1). Scale bar, 50 μm.
