# Supplementary material for: Improved survival of porcine acute liver failure by a bioartificial liver device implanted with induced human functional hepatocytes
Source: Cell Res. 2016 Jan 15;26(2):206–16. doi: 10.1038/cr.2016.6 (PMC4746613; doi:10.1038/cr.2016.6)
Supplement: Supplementary information, Figure S2 — Optimized hiHeps show increased hepatic gene expression and functions [file cr20166x2.pdf]

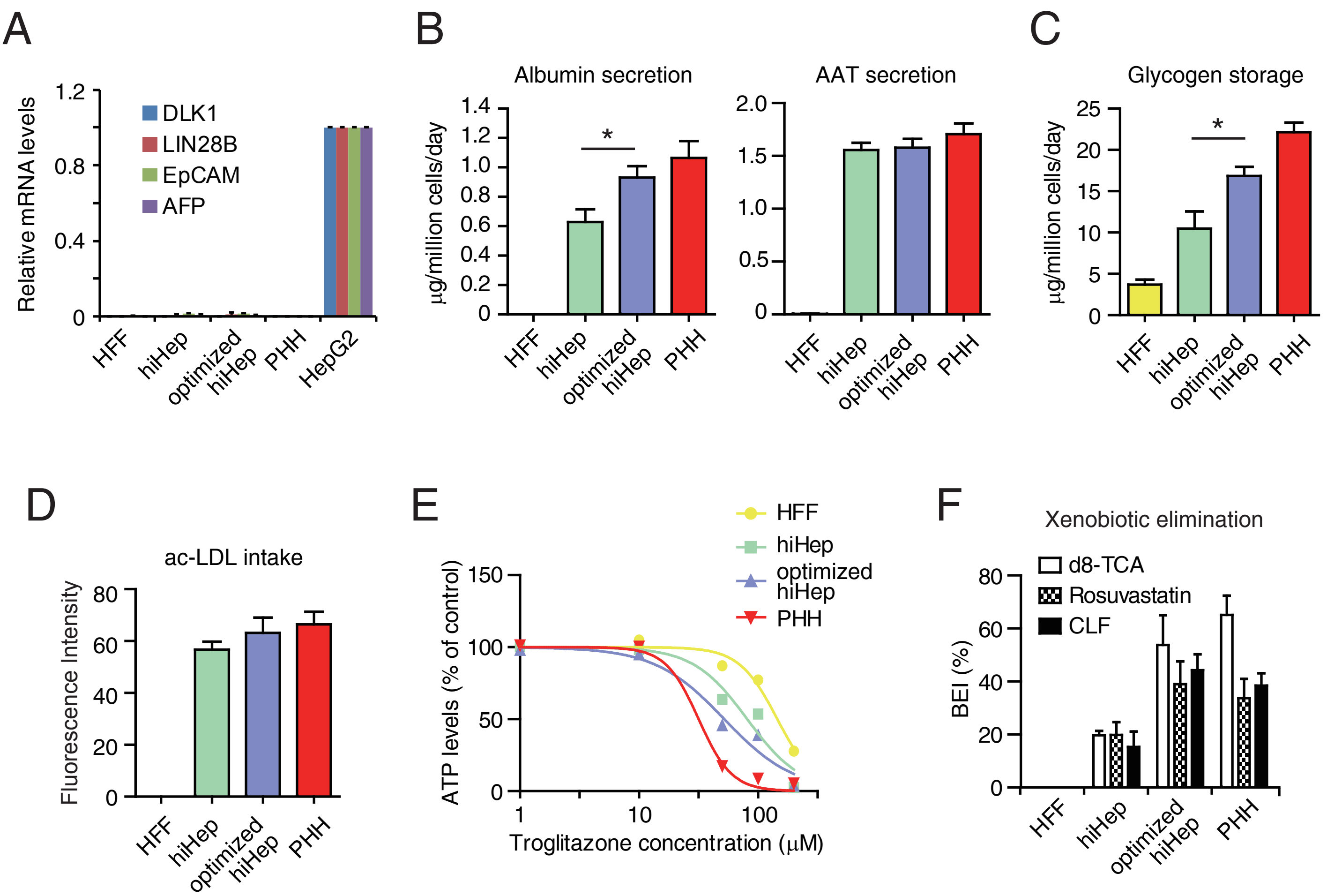


Supplemental Figure 2

**Figure S2. Optimized hiHeps show increased hepatic gene expression and functions**

**A**, Immature marker genes were determined by q-PCR, including delta-like 1 homolog (DLK1), lin-28 homolog B (LIN28B), epithelial cell adhesion molecule (EpCAM), $\alpha$-fetoprotein (AFP). **B**, Albumin and AAT secretion of hiHep cells were measured in culture medium by ELISA. **C**, Glycogen storage in optimized hiHep cells were measured quantitatively. **D**, Intake of acetylated low density lipoprotein labeled with the fluorescent probe 1,1'-dioctadecyl-3,3,3',3'-tetramethyl-indocarbocyanine perchlorate (DiI-ac-LDL) in optimized hiHeps was measured by Operetta. **E**, ATP levels of optimized hiHep cells were measured by the CellTiter-Glo assay during exposure to Troglitazone. **F**, Biliary excretion capabilities of hiHep cells were measured by clearance of D8-taurocholic acid (D8-TCA), Rosuvastatin and cholyl-lysyl-fluorescein (CLF). *, $P<0.05$, *t*-test.
