# Supplementary material for: Improved survival of porcine acute liver failure by a bioartificial liver device implanted with induced human functional hepatocytes
Source: Cell Res. 2016 Jan 15;26(2):206–16. doi: 10.1038/cr.2016.6 (PMC4746613; doi:10.1038/cr.2016.6)
Supplement: Supplementary information, Figure S3 — Stability of hiHep cells during the large-scale expansion [file cr20166x3.pdf]

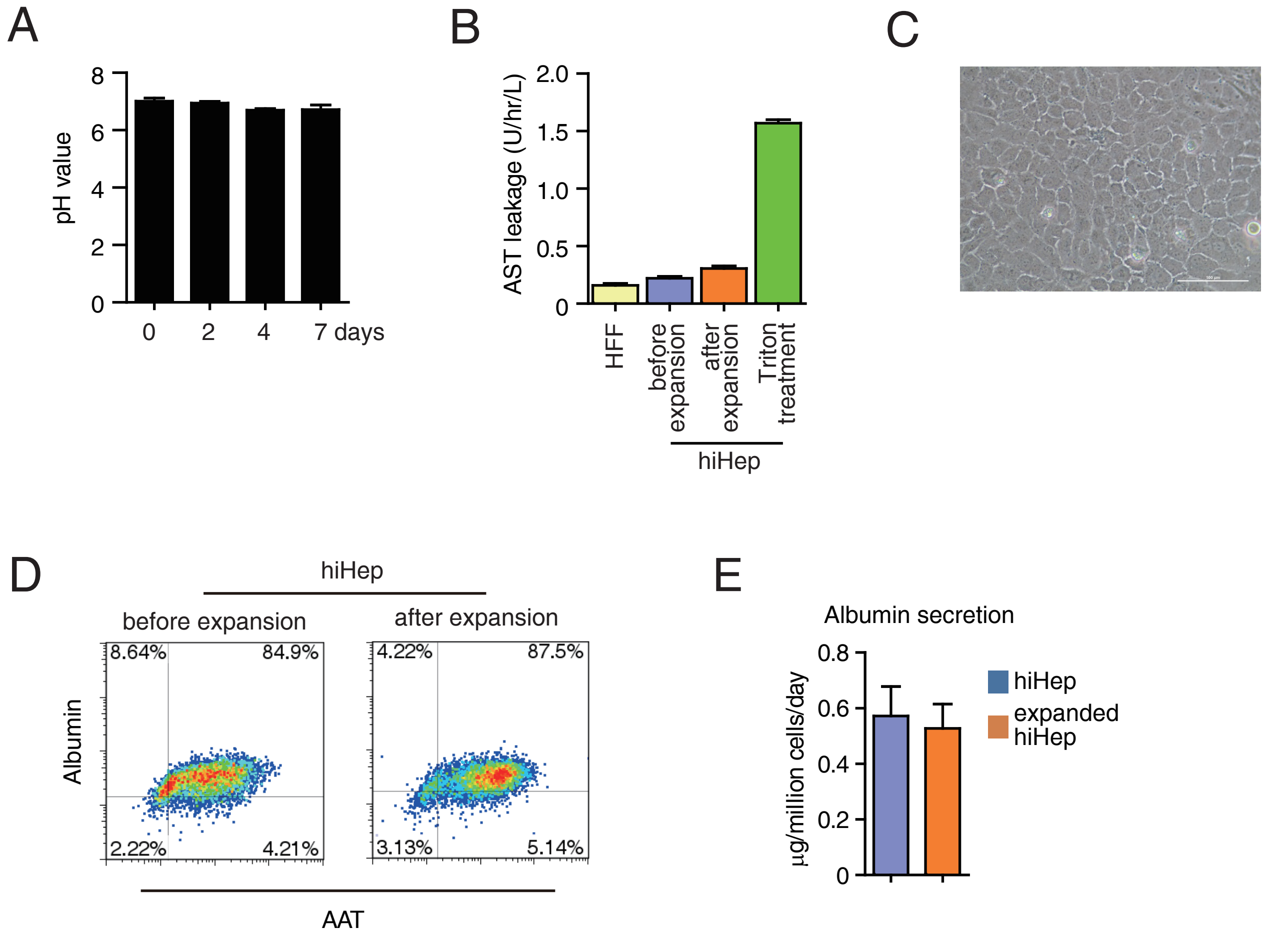

Supplemental Figure 3

Figure S3. Stability of hiHep cells during the large-scale expansion

A, The pH values of hiHep culture media were measured during the large-scale expansion in Hyperflasks. **B**, Aspartate aminotransferase (AST) leakage from hiHeps were measured before and after expansion. **C**, hiHeps after expansion showed homogeneous epithelial morphology. **D**, hiHeps after expansion displayed high percentage of Albumin and AAT double positive cells as measured by flow cytometry. **E**, Albumin secretion of expanded hiHeps was measured by ELISA.
