# Supplementary material for: Improved survival of porcine acute liver failure by a bioartificial liver device implanted with induced human functional hepatocytes
Source: Cell Res. 2016 Jan 15;26(2):206–16. doi: 10.1038/cr.2016.6 (PMC4746613; doi:10.1038/cr.2016.6)
Supplement: Supplementary information, Figure S4 — Consistency of large-scale-expanded hiHeps between different batches [file cr20166x4.pdf]

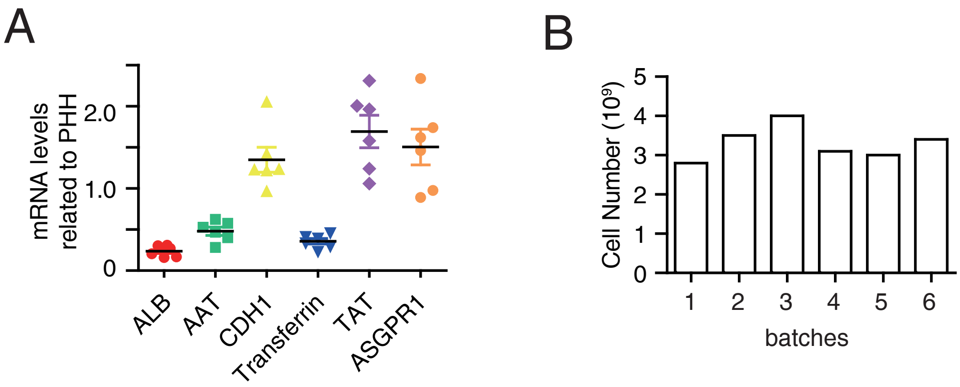

Supplemental Figure 4

Figure S4. Consistency of large-scale-expanded hiHeps between different batches

A, Hepatic gene expression levels of large-scale-expanded hiHeps in different batches were measured by q-PCR. Gene expression levels are normalized to those in PHH. **B**, hiHeps cell numbers produced in different large-scale expansion batches.
