# Supplementary material for: Improved survival of porcine acute liver failure by a bioartificial liver device implanted with induced human functional hepatocytes
Source: Cell Res. 2016 Jan 15;26(2):206–16. doi: 10.1038/cr.2016.6 (PMC4746613; doi:10.1038/cr.2016.6)
Supplement: Supplementary information, Figure S5 — The BAL support system [file cr20166x5.pdf]

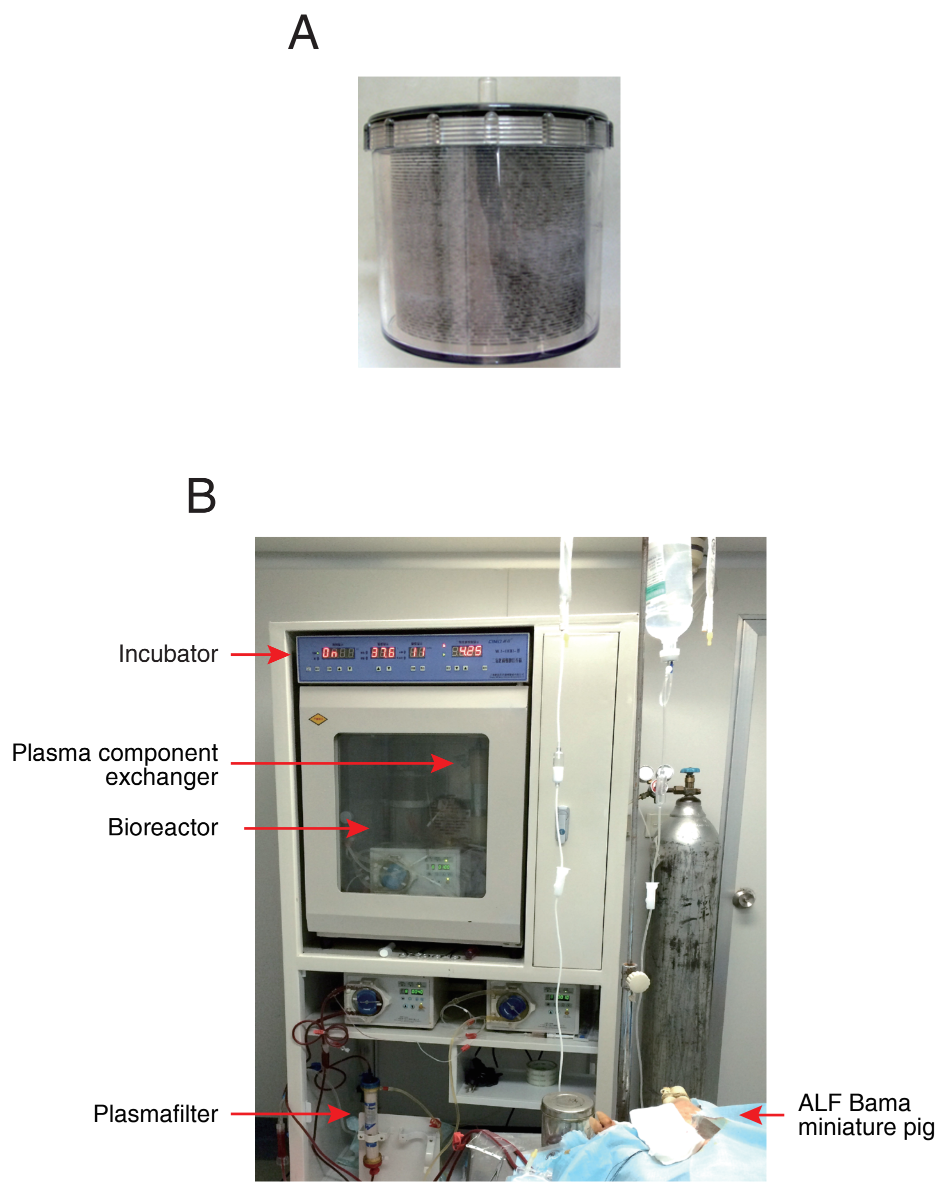

Supplemental Figure 5

Figure S5 The BAL support system

A, Photo of a multi-layer bioreactor of the homemade BAL support system. **B**, Photo of an ALF Bama miniature pig receiving the hiHep-BAL support system treatment.
