# Supplementary material for: Improved survival of porcine acute liver failure by a bioartificial liver device implanted with induced human functional hepatocytes
Source: Cell Res. 2016 Jan 15;26(2):206–16. doi: 10.1038/cr.2016.6 (PMC4746613; doi:10.1038/cr.2016.6)
Supplement: Supplementary information, Figure S6 — Stability of hiHeps in the bioreactor [file cr20166x6.pdf]

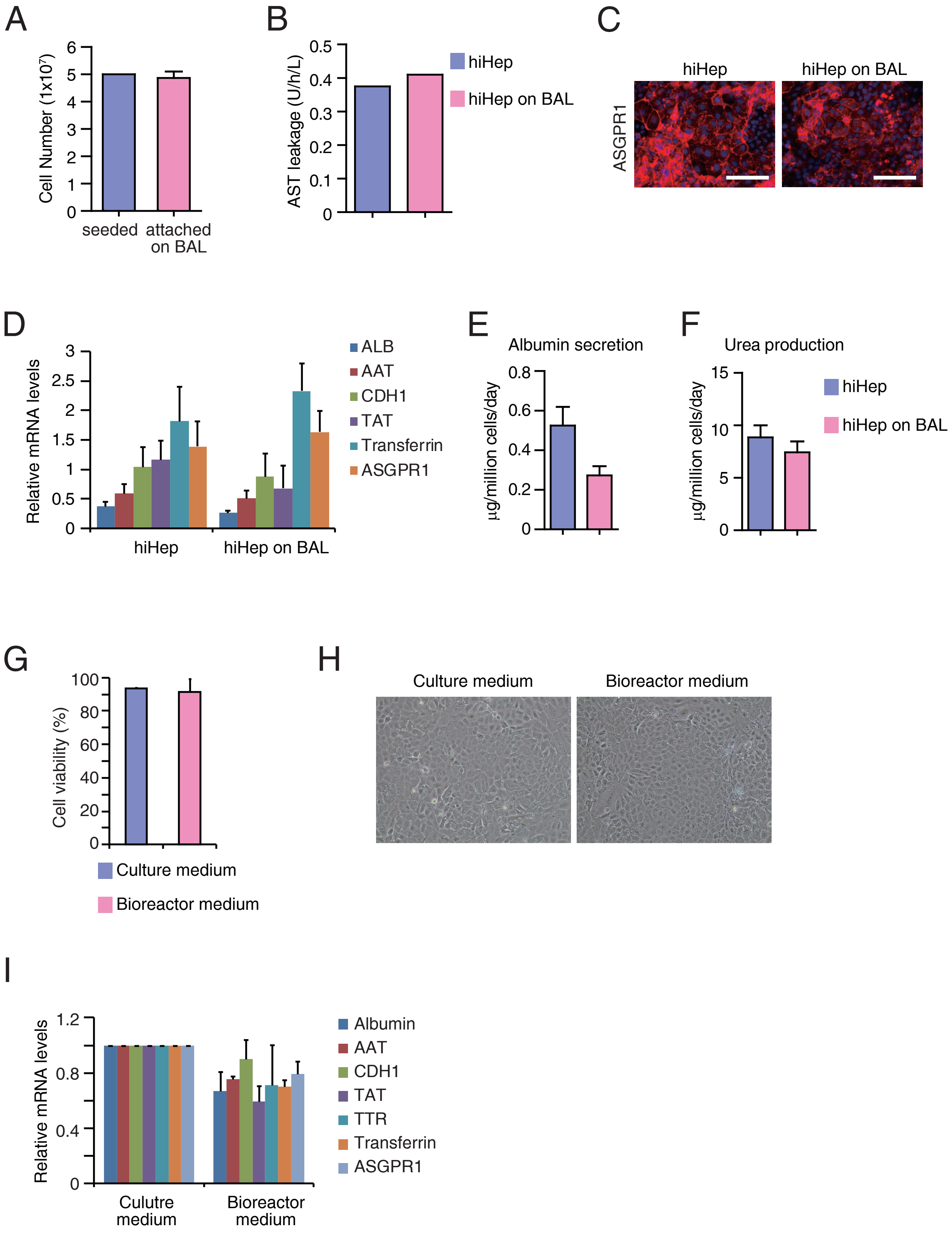

Supplemental Figure 6

Figure S6 Stability of hiHeps in the bioreactor

A, hiHeps attached efficiently to the polycarbonate plate of the bioreactor after seeding. Cell numbers were measured before and after cell adhesion. **B,** Aspartate aminotransferase (AST) leakage was determined after hiHeps were cultured on the polycarbonate plate for 24 hours. **C**, hiHeps remained proper expression and location of ASGPR1, a marker for mature hepatocytes, after attached to the polycarbonate plate. **D-E**, hiHep cells maintained hepatic gene expression (**D**), albumin secretion (**E**) after attached to the polycarbonate plate. **F-H,** Bioreactor medium was collected from hiHep-BAL after treating ALF miniature pigs. hiHeps were tolerant to bioreactor medium after culture for 24 hours. hiHeps maintained cell viability (**F**), epithelial morphology (**G**) and hepatic gene expression (**H**) in the bioreactor medium.
