# Supplementary material for: Improved survival of porcine acute liver failure by a bioartificial liver device implanted with induced human functional hepatocytes
Source: Cell Res. 2016 Jan 15;26(2):206–16. doi: 10.1038/cr.2016.6 (PMC4746613; doi:10.1038/cr.2016.6)
Supplement: Supplementary information, Figure S7 — Establish the D-gal-induced ALF model in Bama miniature pigs [file cr20166x7.pdf]

A

D-Gal	Pig weight	Survival time
0.35 g/kg	17 kg	Survived*
0.35 g/kg	21 kg	Survived*
0.40 g/kg	18 kg	2 days
0.40 g/kg	15 kg	Survived*
0.45 g/kg	15 kg	2 days
0.45 g/kg	23 kg	2 days
0.50 g/kg	23 kg	1 day
0.50 g/kg	15 kg	2 days

B

	Before D-gal	D-gal dosage#			
		0.35 g/kg	0.4 g/kg	0.45 g/kg	0.5 g/kg
ALT (U/L)	60.81±15.24	111.85±39.24	703.10±421.15	185.85±8.56	470.90±445.34
AST (U/L)	112.45±54.94	1000.45±297.62	1346.20±483.10	1711.00±898.03	1377.20±718.14
Ammonia (μM)	55.38±24.51	83.00±1.41	97.50±34.65	66.00±9.90	89.00±2.83
TBIL (μM)	0.46±0.41	16.25±19.02	34.95±9.40	20.50±7.35	22.00±20.79
ALB (g/L)	36.05±3.97	32.25±0.21	40.35±1.77	40.15±0.21	36.35±0.35
LDH (U/L)	722.75±82.25	1325.00±229.10	5581.50±4103.34	1613.00±380.42	1417.50±532.45
PT (s)	11.13±0.45	27.55±0.64	31.40±8.49	17.80±3.11	N.A.

C

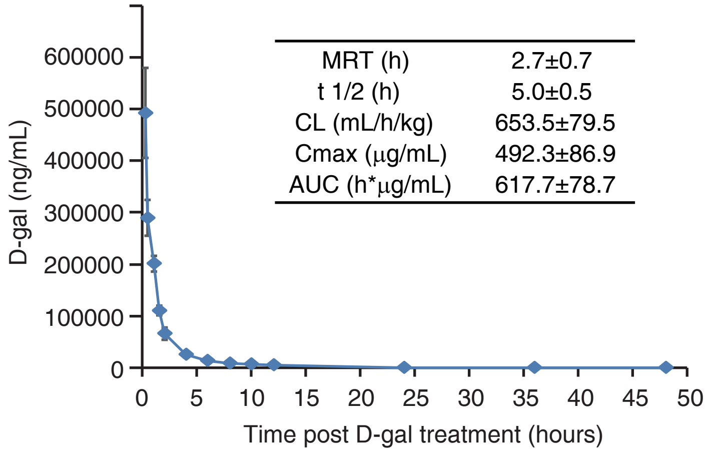

MRT (h)	2.7±0.7
t 1/2 (h)	5.0±0.5
CL (mL/h/kg)	653.5±79.5
Cmax (μg/mL)	492.3±86.9
AUC (h*μg/mL)	617.7±78.7

Supplemental Figure 7

Figure S7 Establish the D-gal-induced ALF model in Bama miniature pigs

A, Bama miniature pigs at the bodyweight around 15-25 kilogram were used in the experiments. Different doses of D-gal were used in induction of acute liver failure in pigs. Survival time was listed. *: All survived animals were sacrificed at day 7. **B,** Serum levels of alanine aminotransferase (ALT), aspartate aminotransferase (AST), ammonia, total bilirubin (TBIL), Albumin, lactate dehydrogenase (LDH) and prothrombin time (PT) were measured by automatic biochemistry analyzer. #: All blood biochemical parameters were measure before D-gal treatment and 1 day after D-gal treatment. **C**, D-gal concentrations in the plasma of Bama minipigs (n=3) were measured by LC-MS/MS at 0.25, 0.5, 1, 1.5, 2, 4, 6, 8, 10, 12, 24, 36 and 48 hours after 0.4 g/kg D-gal administration. 1 Bama minipig was found dead around 40 hours, and the other 2 were sacrificed at 48 hours. Mean residence time (MRT), terminal half-life in plasma (t1/2), clearance (CL), maximum plasma concentration (Cmax), area under the concentration-time curve over the time interval from 0 extrapolated to last measured time (AUC) were calculated and shown.
