# Supplementary material for: Improved survival of porcine acute liver failure by a bioartificial liver device implanted with induced human functional hepatocytes
Source: Cell Res. 2016 Jan 15;26(2):206–16. doi: 10.1038/cr.2016.6 (PMC4746613; doi:10.1038/cr.2016.6)
Supplement: Supplementary information, Figure S8 — Determine the cell number of hiHeps for hiHep-BAL [file cr20166x8.pdf]

A

hiHep number	Pig weight	Survival time*
$1X10^9$	25 kg	2.5 days
$1X10^9$	23 kg	2 days
$1.5X10^9$	18 kg	4 days
$2.6X10^9$	23 kg	Survived
$2.8X10^9$	17 kg	Survived

*: All survived animals were sacrificed at day 7.

B

	hiHep number	Days post D-gal injection							
		Before	1	2	3	4	5	6	7
ALT (U/L)	$1X10^9$	41.3	135.6	765.9	743				
	$1.5X10^9$	54.6	318.2	731.5	604.4				
	$\sim3.0X10^9$	55.65	145.75	906.25	656.25	478.95	370.8	284.05	229.15
AST (U/L)	$1X10^9$	28.2	1761.25	1222.9	3316.4				
	$1.5X10^9$	24.8	2992	4772.5	1682.8				
	$\sim3.0X10^9$	19.9	497.85	3140.75	1617.85	584.05	332.05	205.95	143.3
Ammonia (μM)	$1X10^9$	14.5	81.5	119	300				
	$1.5X10^9$	67	153	500	493				
	$\sim3.0X10^9$	65	150.5	163.5	89	419.5	223.5	57	45
TBIL (μM)	$1X10^9$	0.35	23.2	56.4	72.3				
	$1.5X10^9$	0.4	20.7	59.1	93.7				
	$\sim3.0X10^9$	0.6	22.35	53.8	45	46.35	29.6	8.6	2.6
TBA (μM)	$1X10^9$	24.65	342.1	379.2	384				
	$1.5X10^9$	13.3	310.7	332.6	347.2				
	$\sim3.0X10^9$	16.45	359.6	358.95	305.1	215.15	197.6	128.05	45.55
ALB (g/L)	$1X10^9$	33.9	28.8	30.2	30.7				
	$1.5X10^9$	35.6	28.3	32.7	32.5				
	$\sim3.0X10^9$	40.5	34.95	31.85	31.75	32.75	31.7	32.75	32.95
LDH (U/L)	$1X10^9$	556.5	1167.5	3841	2912				
	$1.5X10^9$	657	2466	8655	4938				
	$\sim3.0X10^9$	490.5	821.5	2940.5	3437	2285.5	1782	1586.5	1451.5

Supplemental Figure 8

Figure S8 Determine the cell number of hiHeps for hiHep-BAL

A, ALF was induced in Bama miniature pigs by D-gal at 0.35 g/kg. Different numbers of hiHeps were implanted to hiHep-BAL to treat the ALF pigs. Survival time was listed. *: All survived animals were sacrificed at day 7. **B,** Serum levels of alanine aminotransferase (ALT), aspartate aminotransferase (AST), ammonia, total bilirubin (TBIL), total bile acids (TBA), Albumin and lactate dehydrogenase (LDH) were measured by automatic biochemistry analyzer. Data are shown as the mean.
