# Supplementary material for: Improved survival of porcine acute liver failure by a bioartificial liver device implanted with induced human functional hepatocytes
Source: Cell Res. 2016 Jan 15;26(2):206–16. doi: 10.1038/cr.2016.6 (PMC4746613; doi:10.1038/cr.2016.6)
Supplement: Supplementary information, Figure S9 — Characterization of hiHep-BAL-treated ALF pigs [file cr20166x9.pdf]

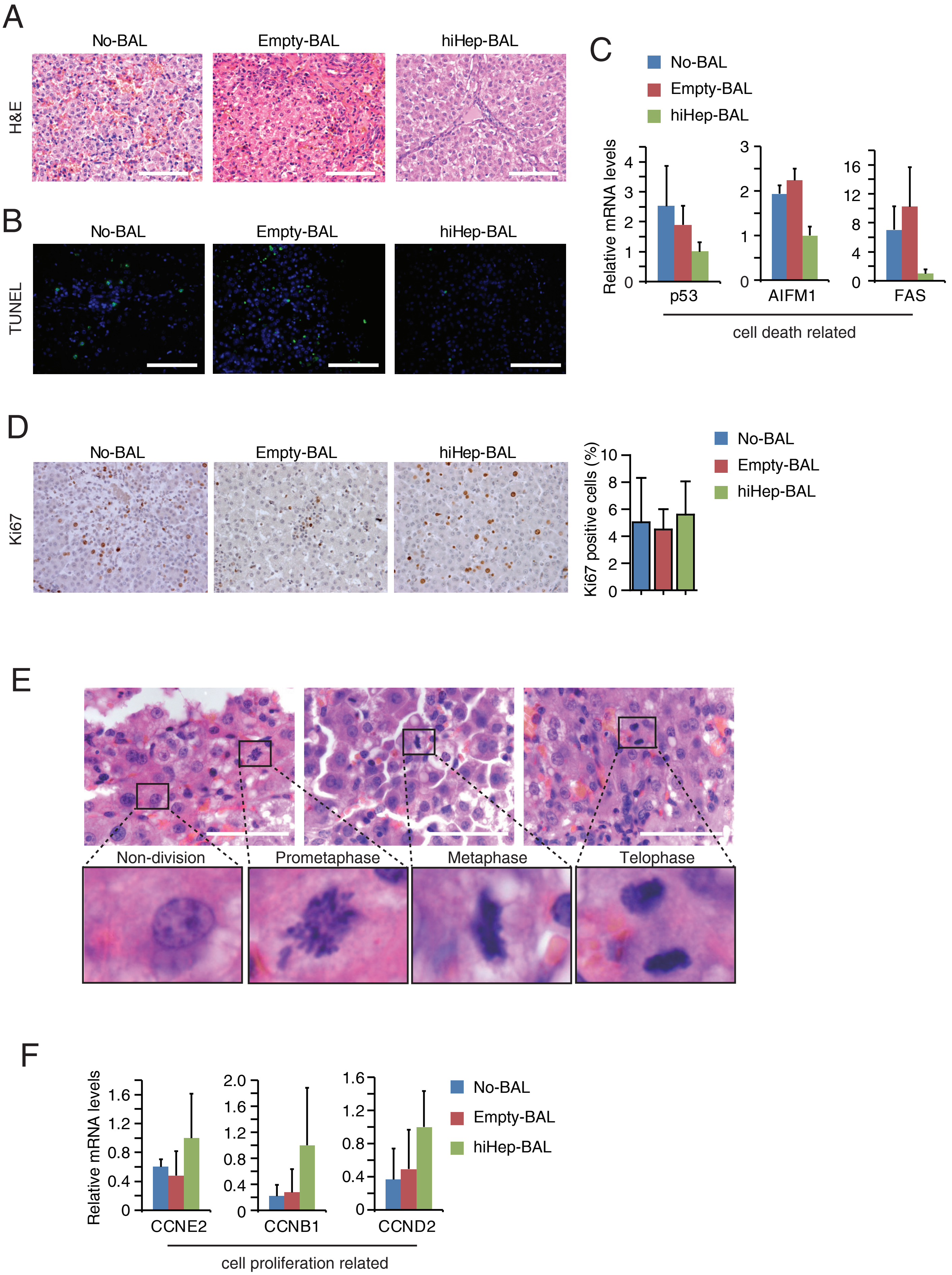

Supplemental Figure 9

Figure S9 Characterization of hiHep-BAL-treated ALF pigs

A, Liver sections of ALF miniature pigs were measured by H&E staining. Note the cell death, hemorrhage and inflammation in No-BAL and Empty-BAL-treated pig livers. Livers of No-BAL and Empty-BAL groups were collected at day 2 or 3 and livers of hiHep-BAL group were collected at day 7 after D-gal treatment. **B**, Cell death was further confirmed by Terminal deoxynucleotidyl transferase dUTP nick end labeling (TUNEL) staining. TUNEL positive cells were stained as green dots overlapped with nuclei. **C,** Expression levels of cell death-related genes were determined in these livers by q-PCR, including p53, apoptosis-inducing factor mitochondrion-associated 1 (AIFM1) and Fas cell surface death receptor (FAS). **D,** Ki67 staining of proliferating hepatocytes. Livers of No-BAL and Empty-BAL groups were collected at day 2 or 3 after D-gal-induced acute liver failure. Livers of hiHep-BAL group were collected at day 7. Ki67 positive cells were quantified. **E**, Histological analyses of proliferating hepatocytes in hiHep-BAL-treated pigs at day 7. The hepatocytes at non-division and different division phases are shown at high magnification. Scale bar, 50 μm. **F**, Expression levels of proliferation-related genes were measured in livers by q-PCR, including cyclin E2 (CCNE2),cyclin B1 (CCNB1) and cyclin D2 (CCND2).
