# Supplementary material for: Improved survival of porcine acute liver failure by a bioartificial liver device implanted with induced human functional hepatocytes
Source: Cell Res. 2016 Jan 15;26(2):206–16. doi: 10.1038/cr.2016.6 (PMC4746613; doi:10.1038/cr.2016.6)
Supplement: Supplementary information, Data S1 — Supplemental Experimental Procedures [file cr20166x10.pdf]

Supplemental Experimental Procedures

Cell culture and hiHep induction

Human fetal fibroblasts (HFF) were cultured in human fibroblast medium (HFM). HFF was immortalized by infection with SV40 Large T, and hiHep was induced from immortalized human fetal fibroblasts by transduction with FOXA3, HNF1A and HNF4A. hiHep cells were cultured in collagen-coated dishes with hepatocyte maintenance medium (HMM). Details about hiHep culture and induction were described in our previous study(Huang et al., 2014). Additional optimizations were listed in Supplementary Figure 1a. For the enrichment of hiHep cells, cells were treated with 2 mg/ml collagenase IV for 10 min. When fibroblast-like cells were detached from the dish and epithelial hiHep cells still attached to the bottom, we removed the collagenase and washed off fibroblast-like cells once with PBS. The remained hiHep cells were digested with trypsin and passaged in new cultures. Up to 80% of cells were Albumin, AAT double positive hiHeps in the culture after this enrichment. Primary human hepatocytes (PHH) from 3 individuals were purchased from Celsis In Vitro Technologies (Lot number: TLQ, FLO, YJM). TLQ hepatocytes were isolated from 52 year-old female patient, FLO hepatocytes were isolated from 14 month-old male child, YJM hepatocytes were isolated from 47 year-old female patient. Detailed information about the PHHs was provided in the product instructions. PHHs were pooled together at the same number and plated in the density of 1.25 x 10^5 /cm^2. For testosterone clearance assay, we used freshly thawed primary human hepatocytes. For rest other assays, we used PHH plated for 2 days. Institutional ethical committees approved collection and use of human samples.

Large-scale expansion

5×10^6 hiHep cells were seeded into 5 dishes (100 mm) for the first-step of expansion. After expansion for 6 days, $3.5\text{x}10^7$ hiHep cells were harvested and seeded in to 35 dishes (100 mm). 6 days later, the final expansion step was performed in 8 Hyperflasks (Corning) with $2.4\text{x}10^8$ hiHep cells from previous expansion. Finally, about 3×10^9 hiHep cells were harvested from the Hyperflasks and used for the hiHep-BAL treatment. pH values of medium in the Hyperflasks were measured at day 0, 2, 4, 7 by pH meter. Medium of the Hyperflasks was collected every day and nutrition components were measured by HLPC.

BAL system

The BAL system consists of a cell circuit and a blood circuit. The components of BAL system include three roller pumps, a heparin pump, a plasma filter (Sorin Group Italia, Mirandola, Italy), a plasma component separator (Kawasumi Laboratories Inc, Tokyo, Japan), and a multi-layer flat-plate bioreactor with polycarbonate scaffolds. As previously reported(Han et al., 2012; Shi et al., 2011), the membrane within the plasma component separator is made from ethylene vinyl alcohol copolymer resin with excellent biocompatibility. The pore size of membranes is 10 nm (~200 KD). The membrane molecular weight cutoff showed high performance in decrease IgG, IgM, deposition of which may reduce the severe xenoreactive antibody response and effectively remove target substance as well(Han et al., 2012; Shi et al., 2011).

As reported(Shi et al., 2012) and shown in Figure S5, the multi-layer bioreactor consists of a hollow column stent, and a stack of 65-layer round flat plates, all of which are made of polycarbonate. The channel height between neighboring plates is maintained at 0.5 mm with the spacers attached to the bottom of each plate. The hiHep cells were implanted into the bioreactor through the eyelets by a peristaltic pump (JHBP-2000B, Guangzhou, China). The height of the bioreactor is about 10 cm, and the effective volume is 480 ml. Freshly harvested hiHep cells were transported in HMM. The whole bioreactor was incubated at 37 ^{o}C and 5% CO_2 overnight until the cells were adhered to the surface of plates and ready for the BAL treatment.

Miniature pig surgery

Chinese Bama miniature pigs (15-25 kg, 4-5 months of either sex)(Liu et al., 2008) were purchased from the Laboratory Animal Center of the Affiliated Drum Tower Hospital of Nanjing University Medical School. All animal procedures were performed according to institutional and national guidelines and approved by the Animal Care Ethics Committee of Nanjing University and Nanjing Drum Tower Hospital. We ordered a pig for the hiHep-BAL treatment each time when hiHep cells were ready. In between two hiHep-BAL treatments, we performed the No-BAL and Empty-BAL control treatments when there were cages and surgery room available for us. To reduce handling complexity, we changed between No-BAL and empty-BAL treatments every two pigs and placed the control treatments randomly.

Pigs in all groups were anesthetized by propofol (0.1-0.2mg/kg/min) and

intravenously injected with D-galactosamine, and baseline blood sample was collected. 24 hours after D-gal injection, animals in Empty-BAL and hiHep-BAL group were anesthetized and 1875 U heparin was injected into artery and vein separately. Then the pigs were catheterized to BAL system via internal carotid artery and internal jugular vein by Seldinger technique as figured. The flow rates of three perfusion cycles in the BAL system were set as follows: 40 ml/min in whole blood perfusion cycle, 10 ml/min in plasma separation cycle, and 100 ml/min in bioreactor cycle. We tried to maintain the stable body temperature during the surgery. The operation room was kept at constant 25 ^{o}C. All ALF pigs were covered with a blanket throughout the surgery to avoid temperature loss. Also, we used a 37 ^{o}C water bath heater in the external circulation to warm the blood before it flew back to the body.

According to our previous studies(Han et al., 2012; Shi et al., 2012; Shi et al., 2011), we chose to treat ALF pigs for 3 hours. During the treatment, heparin was keep injected at 8 U/min, and the pig was anesthetized and put on a respirator to maintain breathing. Animal also received 500 ml saline during treatment to ensure hydration. After the BAL treatment, pigs awoke and were sent back to the cages. Blood samples were collected before and after BAL treatment. And every day afterwards, pig was clinical observed and blood sampled till the animals were sacrificed at 7th day post D-gal injection which is the endpoint of the study. All the animals were kept till they were dead. Dying pigs received humane care. For those survived the critical point of ALF, we euthanized the survived pigs at day 7. All the animal’s liver was collected for H&E, Ki67 staining and q-PCR at the end. Blood samples were used for measurement

of alanine transaminase (ALT), aspartate aminotransferase (AST), ammonia, total bilirubin (TBIL), Albumin, prothrombin time (PT), lactate dehydrogenase (LDH), and cytokine measurements.

Pharmacokinetic analysis

3 Bama miniature pigs were used for D-gal pharmacokinetics study. 3 minipigs were nesthetized by propofol (0.1-0.2 mg/kg/min) and intravenously injected with D-gal at the dosage of 0.4 g/kg. 5 ml Blood samples for determination of the D-gal concentration were taken before the drug administration, and 3 ml at 0.25, 0.5, 1, 1.5, 2, 4, 6, 8, 10, 12, 24, 36, 48 hours after drug administration. Blood was drawn into heparinized tubes, spun immediately and plasma was decanted. The plasma was frozen until processed.

An aliquot of 20 μL sample was protein precipitated with 80 μL IS, the mixture was vortex-mixed well and centrifuged at 13000 rpm for 15 min, 20-25°C. 8 μL supernatant was injected for ultra-performance liquid chromatography coupled with a mass spectrometer (LC-MS/MS) analysis. The sample preparation, LC-MS/MS (API 4000) processes were executed in WuXi AppTec.

RNA extraction Polymerase Chain Reaction, PCR

For most experiments, total RNA was isolated from cells by Trizol (Invitrogen). For RNA extraction from formalin-fixed-paraffin-embedded (FFPE) tissues, RNA was extracted using RNeasy FFPE Kit (Qiagen).

A total of 1 μg RNA was reverse transcribed into cDNA with M-MLV Reverse Transcriptase (Promega) according to the manufacturer’s instructions. Quantitative

PCR (qPCR) was performed with SYBR Premix Ex Taq (TaKaRa) on an ABI StepOne Plus real-time PCR system (Applied Biosystems). Primer sequences will be provided upon request. All qPCR data were performed with at least 2 repeats.

Immunofluorescent staining and flow cytometry analysis

Details about immunofluorescent staining steps are described in our previous works(Huang et al., 2011; Huang et al., 2014). Briefly, cells were fixed by 4% paraformaldehyde (PFA) for 15 min at room temperature and then permeabilized by 0.3% Triton X-100 for 15 min. Cells were washed with PBS three times. After being blocked for by 3% BSA in PBS for 60 min at room temperature, cells were incubated with primary antibodies at 4 ^{o}C overnight, washed three times with PBST, and then incubated with appropriate secondary antibody for 60 min at room temperature in dark. Nuclei were stained with DAPI (Sigma) for 5 min. Antibodies used for immunofluorescent staining are as follows: goat anti-human-Albumin (Bethyl Laboratories, 1:100), rabbit anti-human-α-1-antitrypsin (NeoMarkers, 1:200), rabbit anti-human-CDH1 (GeneTex, 1:100), rabbit anti-human-ZO-1 (GeneTex, 1:100), goat-anti-human-ASGPR1 (Santa Cruz, 1:100), Cy3-conjugated donkey anti-goat IgG (Jackson Lab, 1:1000), Cy5-conjugated donkey anti-rabbit (Jackson Lab, 1:500).

For flow cytometry analysis, cells were harvested and washed once in PBS solution (Hyclone) with 1% BSA, and then fixed by 4% PFA for 15 min and permeabilized by 0.3% Triton X-100 for 15 min. After that, cells were incubated with Albumin and α-1-antitrypsin (AAT) antibodies for 30 min at room temperature, and then incubated

with the secondary antibodies for 15 min in dark at room temperature. After incubation, cells were washed twice and analyzed by the Calibur Flow Cytometer (Becton Dickinson). Antibodies for flow cytometry are as follows: Mouse anti-Albumin (R&D, 2.5 $\mu g/10^6$ cells), Rabbit anti-AAT (NeoMarkers, 1:200). goat anti-mouse IgG-PE (Santa Cruz, 1:500), Cy5-conjugated donkey anti-rabbit (Jackson Lab, 1:500). Data were analyzed with FlowJo software (Tree Star).

ELISA, assays for glycogen storage and intake of ac-LDL

To determine the secretion of human Albumin and α-1-Antitrypsin, supernatants of cell culture were collected after 48 hours culture from HFF, hiHep, optimized hiHep and expanded hiHep. PHH were seeded on 12-well plates for 12 hours, and then maintained in HMM for 48 hours until collection of supernatants. For porcine model experiments, animal serum and bioreactor medium were collected. The amounts of Albumin and α-1-Antitrypsin were measured using human-specific Albumin ELISA Quantitation Set (Bethyl Laboratory) and the human α-1-Antitrypsin ELISA kit (Bethyl Laboratory) according to the manufacturer's instructions. The amounts of porcine IL1β, IL6, IL8, IL10 and TNFα in pig's serum were measured using porcine-specific ELISA Quantitation Set (R&D Systems) according to the manufacturer's instructions. Glycogen storage of hiHep, optimized hiHep, HFF and PHH were determined by Glycogen Assay Kit (Abnova). For the intake of ac-LDL, cells were incubated with DiI-ac-LDL (Invitrogen) for 6 hours, and then measured quantitatively by Operetta (PerkinElmer).

Ammonia elimination assays.

To determine the ammonia elimination abilities, cells were incubated in HMM supplemented with 3 mM NH_4Cl. Supernatant was collected at 0.5 h and 24 h after NH_4Cl induction. NH_4^+ concentrations were measured using the enzymatic colorimetric assays (Megazyme International, Ireland).

CYP metabolism and biliary excretion assay

For measurement of CYP metabolic activities, HFF, hiHep, optimized hiHep, expanded hiHep cells were cultured for 6 days. On the experimental day, culture medium was removed, and hiHep cells were incubated with 5 μM testosterone (Sigma), the CYP3A substrate in 2 ml incubation medium. hiHep cells were incubated on an orbital shaker for 0, 30, 60, 120, 240 min at 80-120 rpm for a 6-well plate. To stop the reaction, 300 μl cold methanol was added into 100 μl medium containing the substrate. The supernatants were collected for measurement of indicated productions by LC-MS/MS (LCMS-8030; Shimadzu, Kyoto, Japan). Plated cryopreserved hepatocytes were used as a positive control and HFF cells were used as a negative control.

D8-TCA and Rosuvastatin were analysed by LC/MS/MS (LCMS-8030; Shimadzu, Kyoto, Japan). The amount of CLF was quantified by measuring fluorescence at 492 nm and 536 nm with a Synergy 4 microplate reader (Biotek, Winooski, USA). Biliary Excretion Index (BEI) was calculated as: BEI=(AHBSS-AHBSS(Ca^{2+}

free))/AHBSS×100%.

ATP assay

ATP level was measured using the CellTiter-Glo® Luminescent Cell Viability Assay according to the manufacturer's instruction (Promega, Madison, USA). Briefly, hiHeps and optimized hiHeps were seeded in the 96-well plate with 1×10^4 cells/well for six days while HFF were cultured for 3 days and PHH were attached for 3 hr. Then, these cells were treated with various concentrations of troglitazone. The exposure period was 24 hour. Equilibrate the plate at room temperature for 30 minutes and add 100 μl CellTiter-Glo® Reagent for each well. Mix contents for 2 minutes and incubate at room temperature for 10 minutes. The luminescence of each well was measured by BioTek SYNFRGY4 microplate reader (Biotek, Winooski, USA)

Histology and immunohistochemistry

Liver tissues were collected immediately at the time pigs were died or sacrificed, and fixed overnight with 4% neutral formalin. Tissue sections were stained with haematoxylin (Sigma-Aldrich) and eosin (Sigma-Aldrich) for pathological evaluation. Ki67 staining was performed according to the protocols described previously(Huang et al., 2014). Antibodies used are as following: primary antibodies NCL-Ki67 (Leica Biosystems）, secondary antibody (Cell signaling Technologies). DAB staining (DAKO) and counterstaining of haematoxylin were performed on that basis.

TUNEL staining was performed using the ApoAlertTM DNA fragmentation Assay Kit (BD Sciences).

Statistics

For most statistic evaluation, an unpaired Student's t test was applied for calculating statistical probability in this study. *P* values were calculated by two-tailed test. For survival analysis, the Mantel-Cox log-rank test was applied. Statistic calculation was performed using Statistical Program for Social Sciences software (SPSS, IBM).
